# Decoding Oral Carcinogenesis and Tumor Progression in Whole Cigarette Smoke Exposure: A Systematic Review

**DOI:** 10.7759/cureus.66966

**Published:** 2024-08-15

**Authors:** Jiao Li, Nurhayu Ab Rahman, Suharni Mohamad

**Affiliations:** 1 Pathology, School of Dental Sciences, Universiti Sains Malaysia Health Campus, Kubang Kerian, MYS; 2 Pathology, Changzhi Medical College, Shanxi, CHN; 3 Oral Medicine and Oral Pathology Unit, School of Dental Sciences, Universiti Sains Malaysia Health Campus, Kubang Kerian, MYS; 4 Oral and Maxillofacial Diseases Research Cluster, School of Dental Sciences, Universiti Sains Malaysia Health Campus, Kubang Kerian, MYS

**Keywords:** oral squamous cell carcinoma, signaling pathways, human oral cells, cigarette smoke, oral carcinogenesis

## Abstract

This systematic review aims to highlight the molecular mechanisms by which whole cigarette smoke affects oral carcinogenesis and its progression in human oral cells, based on evidence from original research articles published in the literature. A literature search was conducted using three databases: Web of Science, Scopus, and PubMed from May to June 2024. The articles were screened, and the data were extracted according to the Preferred Reporting Items for Systematic Reviews and Meta-Analysis (PRISMA) guidelines (2020). The included studies were subsequently evaluated using the Systematic Review Center for Laboratory Animal Experimentation (SYRCLE) tool for bias factors. From the 14 included studies, two types of cell lines were frequently utilized: human oral mucosal epithelial cells or oral squamous cell carcinoma cells. In these cell lines, one of three forms of exposure was applied: cigarette smoke, its extract, or condensate. The mechanism of oral carcinogenesis and tumor progression includes aberrations in the heme metabolic pathway, modulation of miRNA-145, NOD1 and BiP expression, MMP-2, MMP-9, and cathepsin modulation, abnormal TSPO binding, RIP2-mediated NF-κB activation, MZF1-mediated VEGF binding, and activation of the RAGE signaling pathway. In conclusion, cigarette smoke significantly influences the development and progression of oral squamous cell carcinoma, based on the evidence highlighted in human oral cells. While previous studies have focused on specific carcinogens and pathways, this review added to our understanding of the overall impact of whole cigarette smoke on oral carcinogenesis at the molecular and cellular levels.

## Introduction and background

Oral squamous cell carcinoma (OSCC) is the most prevalent type of head and neck squamous cell carcinoma (HNSCC), ranked as the sixth most common cancer in the world [1]. According to Global Cancer Statistics (2020), lip and oral cavity cancer are highly frequent in South Central Asia (e.g., India, Sri Lanka, and Pakistan) as well as Melanesia (Papua New Guinea) [1]. The primary risk factor for OSCC is tobacco smoking, which is three times more likely in smokers than nonsmokers. Other risk factors include betel quid (BQ) chewing, alcohol consumption, and poor dietary habits lacking fruits and vegetables. Additionally, it is estimated that only 3% of OSCC prevalence is related to high-risk human papillomavirus (HR-HPV) infection, compared to more than 90% of oropharyngeal squamous cell carcinoma (OPSCC) cases [2-5]. Despite breakthroughs in medical technology and treatment protocols, the five-year overall OSCC survival rate remains at 65% [6]. Therefore, it is crucial to understand the evidence-based mechanisms underlying the development of cigarette-related OSCC to implement effective preventive healthcare strategies fully.

According to accumulating evidence, cigarette smoke produces over 5,000 distinct chemical constituents, of which over 60 are carcinogenic [7]. In this regard, the International Agency for Research on Cancer (IARC) has reviewed several carcinogens, including nitrosamines, polyaromatic aromatic hydrocarbon (PAH), aromatic amines, aldehydes, phenols, volatile hydrocarbons, nitro compounds, and other organic and inorganic substances. Among which, nitrosamines mainly comprise N′-nitrosonornicotine (NNN) and 4-(N-Nitrosomethylamino)-1-(3-pyridyl)-1-butanone (NNK), while PAH mainly contains Benzo[a]pyrene (B[a]p), DB[a,l]P: Dibenzo [def,p]chrysene (Dibenzo[a,l]pyrene, DB[a,l]P). Animal or human research provided sufficient evidence for each of the discovered carcinogens. For example, B[a]p, NNK, and NNN are group 1 carcinogens, while DB[a,l]P and acrolein are group 2A carcinogens [8]. It is established that these tobacco-derived carcinogens give rise to the development of lung, oral, nose, larynx, oropharynx, hypopharynx, esophagus, stomach, liver, pancreas, bladder, kidney, and cervical cancers, as well as myeloid leukemia [9-17]. Nicotine, the primary component of cigarette smoke, is also extremely addictive. The ability of nicotine to cause cancer per se has been a subject of debate for several decades [18]. Some studies have demonstrated that nicotine causes cancer in A/J mice and epithelial cells in culture, suggesting that it should be designated as a carcinogen [19-23].

Indeed, research on the specific carcinogenic constituents in cigarettes is essential in understanding the underlying mechanism of cigarette smoke-related oral carcinogenesis, as mentioned in previous studies. However, no study has systematically reviewed the evidence on how whole cigarette smoke affects the molecular mechanisms of oral carcinogenesis or its progression. A number of studies have investigated the role that whole cigarette smoke plays in the development of oral carcinogenesis and OSCC. In oral mucosal epithelial cells, cigarette smoke extract (CSE) has been shown to have the potential to suppress nucleotide-binding oligomerization domain 1 (NOD1) expression and modify the expression levels of critical molecules in the NOD1 signaling pathway. This process facilitates the recruitment and activation of receptor-interacting protein 2 (RIP2), which in turn activates nuclear factor κB (NF-κB) and starts the transcription of downstream genes [24]. CSE can also enhance OSCC cell invasion in a way that is dependent on the advanced glycation end-products (RAGE) activation pathway [25].

The goal of this study was to delineate the molecular mechanism induced by whole cigarette smoke exposure in the development or progression of OSCC using data from human-derived oral cell line studies.

## Review

Materials and methods

A systematic literature search was conducted from May to June 2024 on the Web of Science, Scopus, and PubMed (Medline) databases. The population, intervention, comparator and outcomes (PICO) framework served as the basis for the inclusion and exclusion criteria.

The inclusion criteria were original research articles in the English language that fulfilled the following requirements, namely, (1) P: Studies based on the research on human oral cell lines; (2) I: The cells were cultured and exposed to any form of whole cigarette smoke; (3) C: The availability of a control group against test group for comparison; (4) O: The outcomes that demonstrated the molecular mechanism on oral carcinogenesis or its progression.

The following criteria were used to omit studies: reviews, letters, editorial material, book chapters, conference abstracts, clinical studies, in vivo studies, animal studies, and non-English language articles.

The preliminary search strategies can be seen in Appendix 1.

Additionally, studies were excluded if they lacked sufficient evidence to link cigarette smoke to oral carcinogenesis or the progression of OSCC. For instance, studies where cigarette smoke only caused inflammatory responses or showed no significant difference between control and treatment groups were not considered.

The screening and selection of articles were conducted following the Preferred Reporting Items for Systematic Reviews and Meta-Analyses (PRISMA) guidelines [26]. Both investigators (JL and NAR) double-checked the results. Each study’s title and abstract were reviewed. Subsequently, the identified studies underwent eligibility and cross-referencing screening. All related articles published from the beginning of the database’s records up to June 2024 were considered for inclusion. Endnote v20.4 (Clarivate Plc, Philadelphia, PA) was used for the screening procedure, utilizing its built-in "check for duplicates" feature to eliminate redundant research. The number of duplicate hits in each database and the overall number of hits based on the search outputs were noted. After meeting the inclusion criteria, abstracts were pulled for additional screening before obtaining the full-text papers. 

From each of the included papers, the surname of the first author, the year the study was conducted, the cell line utilized in the investigation, the interventions, and the outcome findings were extracted. Additionally, the corresponding authors of the included studies were contacted directly in case any information was missing.

Study Quality and Risk of Bias Assessment

For animal research, the Cochrane risk of bias tool was adjusted for bias factors unique to animal intervention studies using the Systematic Review Center for Laboratory Animal Experimentation (SYRCLE) tool [27]. Additionally, because there was currently no well-recognized, standardized risk of bias instrument for in vitro research, the SYRCLE tool was also utilized for in vitro cell model investigations [28]. The SYRCLE tool evaluated selection bias (associated with sequence generation, baseline characteristics, and allocation concealment), performance bias, detection bias, attrition bias, reporting bias, and other kinds of bias. The majority opinion determined the total SYRCLE risk of bias tool score, which was classified as high, low, or indeterminate. A minimal risk of bias across all evaluated domains was considered a sign of a high-quality study.

Results

**Figure 1:**
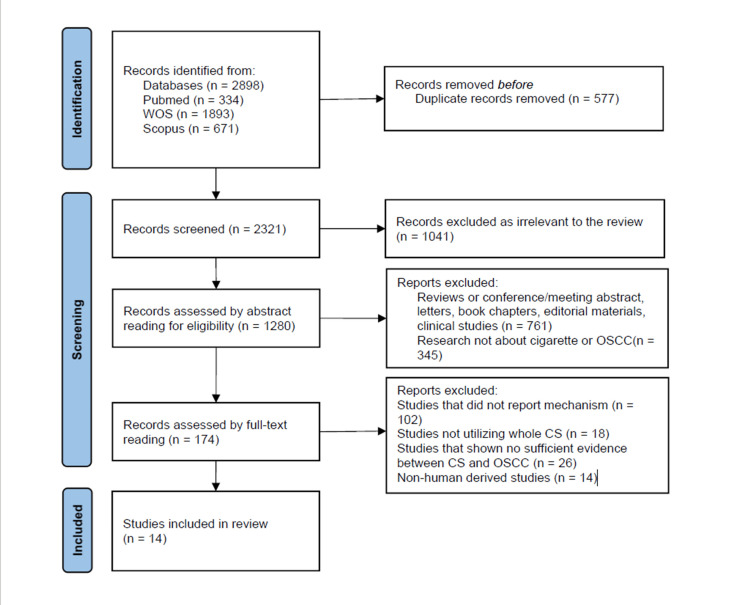
PRISMA 2020 flow diagram for new systematic reviews. Three databases were searched: Web of Science, PubMed, and Scopus.

A total of 2,898 preliminary articles were identified in accordance with the search, selection, and evaluation outlined in the method section (Figure 1).

A total of 14 articles met the inclusion and exclusion criteria and were added to the qualitative synthesis in this review (Table 1).

**Table 1:** Characteristics of the included studies Abbreviation: CS, cigarette smoke; CSE, cigarette smoke extract; CSC, cigarette smoke condensate; NOD1, nucleotide-binding oligomerization-domain 1; miRNA, micro-RNA; MMP, matrix metalloproteinase (MMP); TSPO, translocator protein 18kDa; RIP2, receptor-interacting protein 2; NF-κB, nuclear factor κB; MZF1, myeloid zinc finger 1; VFGF, vascular endothelial growth factor (VEGF); BiP, binding immunoglobulin protein; miR-30a, miRNA-30a (miR-30a); OSCC, oral squamous cell carcinoma; RAGE, receptor for advanced glycation end-products

Author, Year	Type of human-derived cell lines	Form of CS exposure	Outcome
Gao et al., 2015 [24]	Oral mucosal epithelial cells (Leuk-1)	CSE	CSE could inhibit NOD1 expression and affect expression levels of crucial molecules of NOD1 signaling in oral mucosal epithelial cells.
Chapman et al., 2018 [25]	OSCC cells (Ca9-22)	CSE	CSE increased invasion of OSCC cells in a RAGE-dependent manner. Inhibition of RAGE decreases the levels of its signaling molecules, which results in blocking the CS extract-induced invasion.
Gavish et al., 2017 [29]	SCC-15	CS	A rapid decrease in TSPO binding to the high-affinity site induced by exposure to CS
Nagler et al., 2015 [30]	SCC-25 SCC-15	CS	SCC-15 cells showed a 70% (p<0.05) increase in apoptotic levels immediately after 30 min of exposure to CS. Twenty-four hours after 30-min CS exposure a further increase in apoptotic levels to 178% (p<0.05); SCC-15 cells showed a decrease in apoptotic levels immediately after 180-min exposure to CS. SCC-25 cells did not show such CS-related effects.
Nagler et al., 2010 [31]	SCC-25 SCC-15	CS	TSPO binding decreased by 56% and 72%, in the SCC-25 and SCC-15 cell lines, respectively (p<0.05) following CS exposure.
Krayzler et al (b)., 2015 [32]	SCC-25 SCC-15	CS	SCC-15: At 60 and 90 min, increases in the pre-G(1) cell fraction were noted at 118% (p<0.05) and 135% (p<0.01) respectively. The G(2)/M cell fraction was significantly lower following CS exposure. At 90 and 120 min following CS exposure, the G(2)/M fraction levels had declined by 44% (p<0.05) and 34% (p<0.01) respectively. SCC-25: At 90 and 120 min following CS exposure, the pre-G(1) fraction of the cells increased by 230% and 550%, respectively (p<0.01). At 120 min of CS exposure, the fraction of G(2)/M cells was lower by 47% (p<0.05) compared to controls.
Krayzler et al (a)., 2015 [33]	SCC-25 SCC-15	CS	Protein oxidation increased significantly during CS exposure. Carbonyl levels increased six-fold (p<0.001) in both cell lines. Cell viability decrease was time-dependent. Longer CS exposure led to higher cell mortality. At 120 min, SCC-25 cell survival reduction was 43.7%, (p<0.01). Propidium iodide (PI) assay results matched the Trypan blue assay showing a time-dependent cell viability decrease following CS exposure. At 120 min, cell survival reduction was 37% (p<0.05).
Sarkar et al., 2019 [34]	Oral keratinocyte cell (MOE1A)	CSE	CSE alters mitochondrial heme metabolic pathway and initiates cancer progression through modifying cellar biomarkers in oral epithelial cells.
Qian et al., 2022 [35]	OSCC cell lines (HSC-3)	CSE	CSE-induced RIP2 mediated NF-κB activation and caspase-12 upregulation
Krueger et al., 2020 [36]	OSCC cells (HNSCCUM-02T)	CSE	MZF1 binding of VEGF-promoter; directly affects VEGF-gene regulation under CSE exposure.
Pal et al., 2013 [37]	Oral fibroblasts	CSC	Pri-, pre-, and mature miRNA-145 were significantly down-regulated in response to CSC, and this was accompanied by up-regulated expression of MMP-2 and increased migration of fibroblasts compared to untreated controls.
Nagaraj et al., 2007 [38]	686Tu 101A	CSC	Treatment of OSCC cells (686Tu and 101A) with CSC-activated cathepsins B, D, and L in a dose-dependent manner.
Allam et al., 2011 [39]	SCC-25 CAL-27	CSC	Exposure to CSC increased the collagen degrading ability of the SCC-25 by a mechanism involving increased MMP-2 and MMP-9 production.
Chien et al., 2021 [40]	OSCC cells (YD38 and SCC25)	CSC	CSC increased the expression of BiP in time- and dose-dependent manners in YD38 and SCC25 cells; silencing BiP abrogated CSC-induced cell invasion and tumor-associated angiogenesis; CSC increased the expression of BiP in OSCC cells by downregulating miR-30a. BiP promoted invasion and tumor-associated angiogenesis by increasing the production and secretion of VEGF in CSC-exposed OSCC cells.

**Figure 2:**
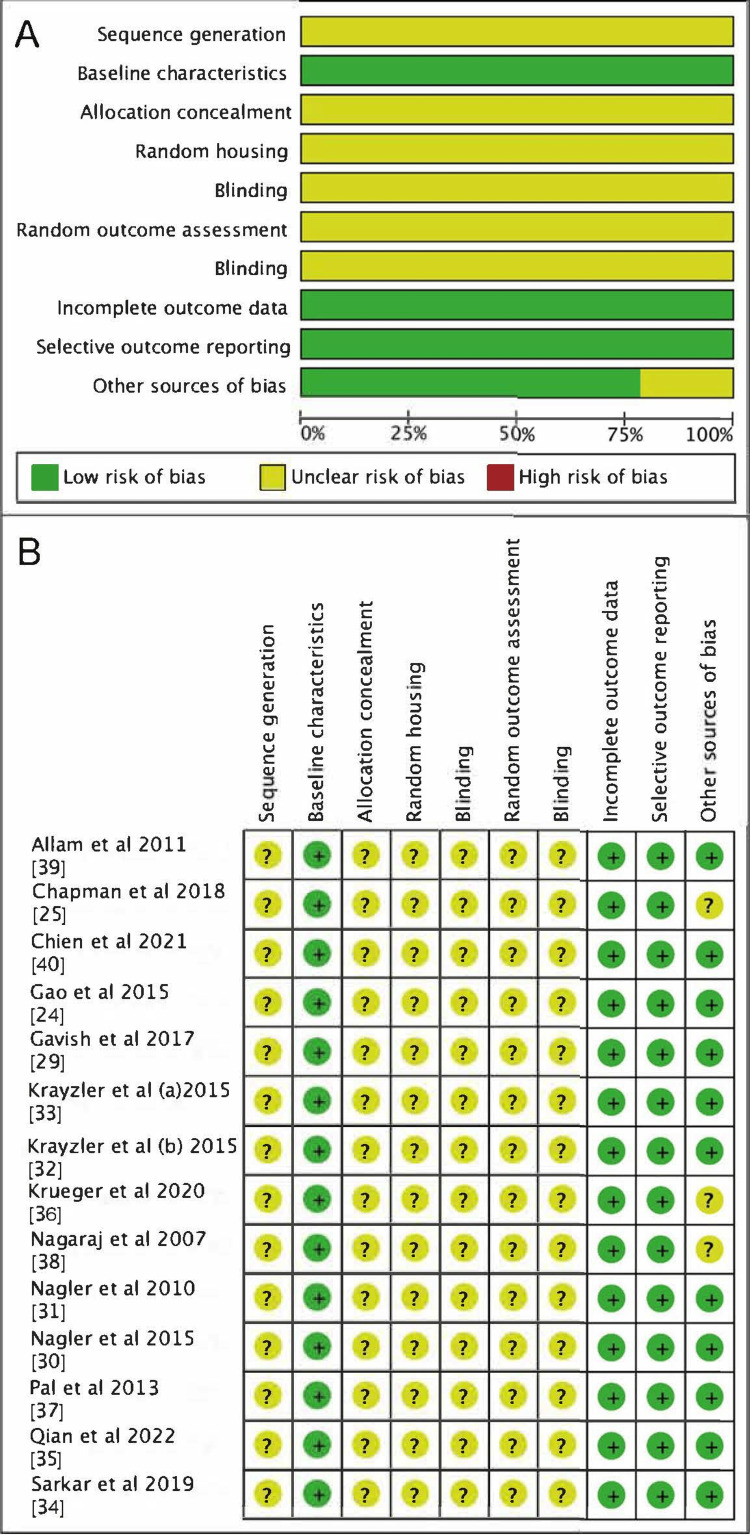
Risk of bias assessed by the Systematic Review Center for Laboratory Animal Experimentation (SYRCLE) guidelines. (A) A graphic summary of the evaluations of the 14 articles that were included. (B) An overview of each article's ratings according to the SYRCLE domains. Some domains displayed unclear risk of bias due to insufficient information available.

The risk of bias evaluation for each of the 14 studies is summarized in Figure 2. No section of any article was considered high risk.

Study Characteristics

The included studies were conducted within the time frame of 2007 to 2022. Out of 14, five studies used whole cigarette smoke exposure [29-33], another five studies used CSE [24, 25, 34-36], and four studies used cigarette smoke condensate (CSC) [37-40]. The cell lines used in the studies were as follows: three studies used normal oral mucosa epithelial cells, i.e., oral keratinocyte cells (MOE1A) [34], oral mucosal epithelial cells (Leuk-1) [24], and oral fibroblasts [37]. The remaining 11 studies utilized OSCC cells to investigate the impact of cigarette smoke on OSCC progression. The most commonly selected cell lines include SCC15, SCC25, CAL-27, HSC-3, Ca9-22, HNSCCUM-02T, 686Tu, and YD38.

The mechanism of oral carcinogenesis and tumor progression demonstrated by these studies includes aberrations in the heme-metabolic pathway, modulation of miRNA-145, NOD1 and BiP expression, MMP-2, MMP-9, and cathepsin modulation, abnormal TSPO binding, RIP2-mediated NF-κB activation, MZF1-mediated VEGF binding, and activation of the receptor for advanced glycation end-products (RAGE) signaling pathway.

Discussion

According to published research, cigarette smoke contains a variety of carcinogenic substances that cause oral cancer. Nevertheless, it is unclear how cancer develops and progresses when human oral cells are exposed to whole cigarette smoke. The most widely accepted mechanism connecting cigarette smoke to the progression of cancer is the production of excess reactive oxygen species (ROS) [41], which raises mitochondrial dysfunction and metabolic activity [42]. The synthesis of heme is connected to the metabolic activity of mitochondria. It has been demonstrated that exposure to CSE leads to increased levels of ROS, initiates heme catabolism-inducing HO-1 gene expression, and triggers the epithelial-mesenchymal transition (EMT) process in oral epithelial cells (MOE1A) via the Nrf2/HO-1 pathway [34]. These incidents could be a factor in the spread of oral cancer. ROS have paradoxical effects on cancer: low ROS levels promote cancer cell proliferation, while high ROS levels induce cell death [43]. This systematic review highlights ROS's role in oral carcinogenesis. High ROS levels reduced cell viability in MOE1A cells over 24 hours and increased apoptosis in SCC-15 cells after 30 minutes and 24 hours of exposure. Surprisingly, SCC-25 cells did not show similar cigarette smoke-related effects [30, 34]. However, Krayzler et al. [33] reported reduced cell survival in SCC-25 cells under cigarette smoke exposure (120 minutes) in the same experimental context as Nagler et al. [30]. This discrepancy may arise from differences in the cell viability test methods used.

Furthermore, cell cycle analysis revealed that cigarette smoke exposure significantly increases the proliferation rate of SCC-15 and SCC-25 cells [32]. This effect is attributed to the 18 kDa translocator protein (TSPO), which significantly influences the proliferation of various cancer cells [29]. TSPO knockdown has been shown to disrupt mitochondrial function in hepatocellular carcinoma (HCC) by increasing ROS production and lowering mitochondrial membrane potential (ΔΨm), which in turn inhibited HCC cell proliferation [44]. When lung cancer cells (H1299) were exposed to cigarette smoke, TSPO expression was upregulated, and a series of biological events leading to cell death were initiated [45]. The knockdown of TSPO can induce apoptosis and inhibit proliferation, as shown in research utilizing anti-sense TSPO knockdown methods to down-regulate TSPO in MA-10 Leydig tumor cells, which resulted in increased tumorigenicity both in vivo and in vitro [46]. In addition, researchers have utilized various techniques for TSPO knockdown and different types of TSPO ligands to reduce apoptotic rates through the mitochondrial apoptosis cascade mechanism [47, 48]. According to Nagler et al. (2010), there was a clear link between lower patient survival rates and increased TSPO expression in oral cancer tissue [31]. However, after cigarette smoke exposure, TSPO binding decreased in the SCC-25 and SCC-15 cell lines [29, 31]. This indicates that cigarette smoke exposure affects TSPO binding differently in specific cell lines. This requires further studies to elucidate the underlying mechanisms and to explore the potential implications for oral carcinogenesis and therapeutic interventions.

As tumors grow beyond a certain size, they require oxygen and nutrients for continued growth, invasion, and metastasis [49]. Angiogenesis is critical in this process, and one important component is the vascular endothelial growth factor (VEGF) [50]. According to the study by Chien et al. (2021), BiP promoted VEGF production and secretion in OSCC cells (YD38 and SCC25) after CSC exposure, mediated by miR-30a. This resulted in tumor invasion and angiogenesis [40]. Another study showed that nicotine, one of the primary cigarette smoke constituents, activated the YAP-TEAD transcriptional complex, which in turn induced BiP expression and caused malignant behaviors such as invasion, migration, and changes in EMT [51]. It was worth noting that MZF1 binding of VEGF-promoter directly influences VEGF-gene regulation in HNSCCUM-02T cells exposed to CSE, and this effect may be more significant in OSCC than in lung cancer cells. [36].

In addition to VEGF, pattern-recognition cell-surface RAGE also shows increased expression during exposure to second-hand smoke [52]. Studies have demonstrated that RAGE plays an important role in the invasion of OSCC [53]. Chapman et al. (2018) reported that CSE could increase the invasion of OSCC cells (Ca9-22) through activation of the RAGE signaling pathway [25]. Specifically, it mediates signaling intermediates Ras, AKT, and NF-κB, which is consistent with findings by Sanders et al. (2017) [54]. Additionally, it regulated matrix metalloproteinases MMP-2, MMP-9, and MMP-14, and upregulation of these resulted in collagen degradation and tumor invasion. Allam et al. (2011) showed a similar result regarding CSC-induced upregulation of MMPs (MMP-2 and MMP-9), which was observed in SCC-25 cells but not in CAL-27 cells [39]. The authors hypothesized that this difference in response could be attributed to the origin of the cell lines: SCC-25, derived from a metastasizing tongue lesion, and CAL-27, derived from a non-metastasizing tongue lesion. Consequently, CSC treatment appears to have a more significant impact on the behavior of metastasizing tongue SCC than non-metastasizing tongue SCC [39]. However, the impact of RAGE signaling following cigarette smoke exposure in Ca9-22 cells derived from human gingival carcinoma remains unclear [25]. Additionally, CSC-induced upregulation of MMP-2 was also detected in oral fibroblasts due to down-regulation of pri-, pre-, and mature miRNA-145 [37]. These findings suggest that the RAGE-MMPs axis and RAGE/ Ras, AKT, and NF-κB pathway may play a pivotal role in cigarette smoke-induced oral carcinogenesis and tumor progression.

Other than MMPs, activation of cathepsins B or L may have been involved in the increased invasion of CSC-treated cells. Administration of these inhibitors prevented cancer cells from invading. It has been shown that cathepsins B and L were crucial in matrix degradation and cell invasion [55-57]. Moreover, cigarette smoke was known to activate TNF-α, probably via cathepsins B mediated TNF-α-induced tumor cell death [38]. Cathepsins B and L were identified as the two main factors that influenced the invasive growth of CSC-treated OSCC cells (686Tu and 101A) [38]. This effect probably resulted from the cytochrome P450 enzymes CYP1A1 and CYP1B1 breaking down carcinogens into compounds that attach to TNF-α receptors [58].

Another pathway implicated in cigarette smoke-induced oral carcinogenesis involves the nuclear factor κB (NF-κB) signaling pathway, where receptor-interacting protein 2 (RIP2) plays a crucial role in regulating inflammatory and cell death processes [59]. RIP2 is also a predictive biomarker and therapeutic target, and it plays a role in the development, spread, and metastasis of various tumor types. [60, 61]. In OSCC cells (HSC-3), cigarette smoke may upregulate the RIP2/caspase-12/NF-κB axis [35]. This finding is in line with earlier research, which found that cigarette smoke increased the expression of RIP2 in mouse lung tissues and RIP2 gene silencing could lessen acute lung injury [62].

In summary, we explore three key mechanisms underlying cigarette smoke-related oral carcinogenesis and progression in human oral cells. First, cigarette smoke exposure triggers increased ROS levels via the HO-1 - EMT - Nrf2/HO-1 pathway, impacting adaptability in cancer cells. Second, TSPO influences cellular proliferation and apoptosis in specific OSCC cell lines, suggesting that TSPO dysfunction could play a role in the development of OSCC. Third, whole cigarette smoke exposure contributes to tumor growth and progression through angiogenesis (VEGF regulation), elevated BiP expression (linked to nicotine activation of the YAP-TEAD complex), and the RAGE signaling pathway (involving intermediates Ras, AKT, and NF-κB). Additionally, MMPs play a role in collagen degradation, while cathepsins mediate cigarette smoke-activated TNF-α. The upregulation of the RIP2/caspase-12/NF-κB axis impacts inflammatory processes and cell death. Consequently, targeting RIP2 pharmacologically may offer a strategy to control the development of cigarette smoke-related OSCC. Further studies are required to determine this approach’s feasibility, assess its efficacy, and ensure its safety in clinical settings.

## Conclusions

We highlighted various mechanisms of cigarette smoke-induced oral carcinogenesis and tumor progression in human oral cells. Based on the evidence, we concluded that cigarette smoke significantly influences the development and progression of OSCC. While previous studies have focused on specific carcinogens and pathways, this review enhanced our understanding of the overall impact of whole CS on oral carcinogenesis at the molecular and cellular levels. However, further research is needed to elucidate specific mechanisms by which cigarette smoke contributes to OSCC development.

## Appendices

Table 2 outlines the preliminary search strategy method utilized in the study. 

**Table 2:** The preliminary search strategy method The search method combined free words, Boolean search strings, and medical topic headings (MeSH keywords) when available.

The preliminary search strategy method
P: ("Squamous Cell Carcinoma of Head and Neck"[Mesh]) OR (“Oral Squamous Cell Carcinoma”[Title/Abstract]) OR (“Oral Cavity Squamous Cell Carcinoma”[Title/Abstract]) OR (“Oral Tongue Squamous Cell Carcinoma”[Title/Abstract]) OR (“Squamous Cell Carcinoma of the Mouth”[Title/Abstract]) OR (“OSCC”[Title/Abstract]) OR ("Mouth Neoplasms"[Mesh]) OR (“Oral Neoplasm”[Title/Abstract]) OR (“Oral Cancer”[Title/Abstract]) OR (“Mouth Cancer”[Title/Abstract])
AND
I: ("Tobacco Products"[Mesh]) OR (“Cigarette*” [Title/Abstract]) OR (“Bidi*” [Title/Abstract]) OR (“Kretek*” [Title/Abstract])) OR (“Cigar*” [Title/Abstract]) OR (“Pipe Tobacco*” [Title/Abstract]) OR (“Cigarillo*”[Title/Abstract]) OR (“Tobacco Product*”[Title/Abstract])
NOT
("Electronic Nicotine Delivery Systems"[Mesh]) OR (“Electronic Cigarettes” [Title/Abstract]) OR (“E-Cig*” [Title/Abstract]) OR (“E-Cigarette*” [Title/Abstract]) OR (“Electronic Cigarette” [Title/Abstract])
NOT
("Tobacco Smokeless"[Mesh]) OR (“Smokeless Tobacco”[Title/Abstract]) OR (“Tobaccos Smokeless”[Title/Abstract])) OR (“Gutka*”[Title/Abstract]) OR (“Gutka Tobacco*”[Title/Abstract]) OR (“Snus”[Title/Abstract]) OR (“Dipping Tobacco*”[Title/Abstract]) OR (“Oral Tobacco”[Title/Abstract]) OR (“Chewing Tobacco*”[Title/Abstract]) OR (“Snuff”[Title/Abstract]) OR (“Mint Snuff”[Title/Abstract])
NOT
("Vaping"[Mesh]) OR (“THC Vaping*” [Title/Abstract]) OR (“E-Cig Use” [Title/Abstract])) OR (“Vape*” [Title/Abstract])) OR (“Nicotine Vaping*” [Title/Abstract])
